# Supplementary material for: Multi-spectral Fluorescent Reporter Influenza A Viruses Allow for in vivo Studies of Innate Immune Function in Zebrafish
Source: bioRxiv. 2023 Nov 2:2023.10.31.564888. Preprint. [Version 1] doi: 10.1101/2023.10.31.564888 (PMC10634972; doi:10.1101/2023.10.31.564888)
Supplement: Supplement 1 [file NIHPP2023.10.31.564888v1-supplement-1.pdf]

bioRxiv preprint doi: https://doi.org/10.1101/2023.10.31.564888; this version posted November 2, 2023. The copyright holder for this preprint (which was not certified by peer review) is the author/funder, who has granted bioRxiv a license to display the preprint in perpetuity. It is made available under aCC-BY 4.0 International license.

SUPPLEMENTAL FIGURES

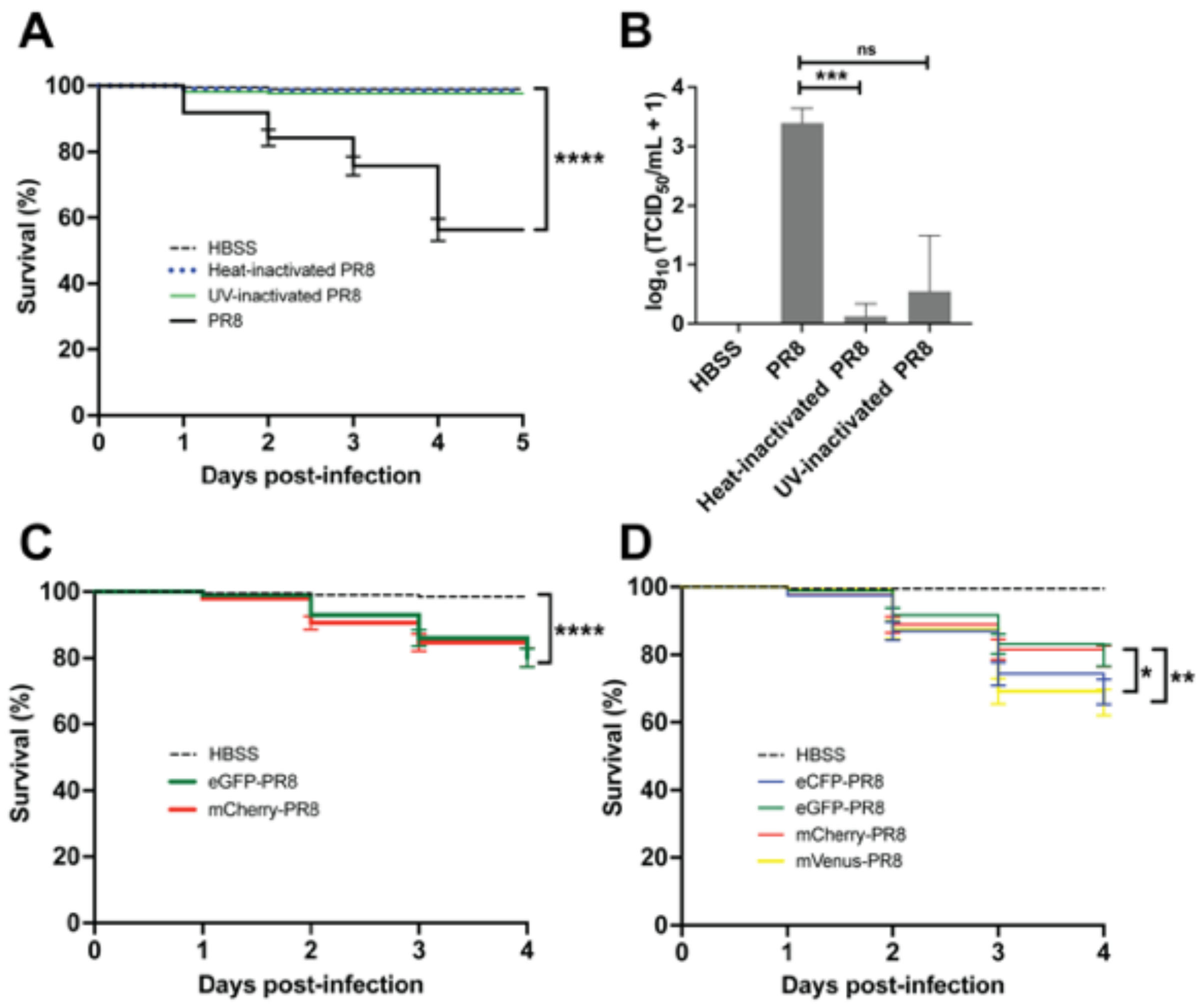

Figure S1: A) Survival of AB larvae injected with live PR8 (8.7 x 10^2 EID_{50}), heat-inactivated PR8, UV-inactivated PR8 or HBSS (vehicle control) at 2 dpf. Live PR8 infection resulted in significantly lower survival than HBSS, heat-inactivated PR8, or UV-inactivated PR8 ($p < 0.0001$). B) Viral burden of larvae at 24 hpi with live PR8 (8.7 x 10^2 EID_{50}), heat-inactivated PR8, and UV-inactivated PR8 at 2 dpf. 24 hpi. Viral burden was different between live PR8 and heat-inactivated PR8 ($p = 0.0001$). C) Survival of larvae was reduced with eGFP-PR8 and mCherry-PR8 infection compared to HBSS controls ($p < 0.0001$). D) Survival of Color-flu infected larvae was lower with eCFP-PR8 ($p = 0.0288$) and mVenus-PR8 ($p = 0.0063$) infected larvae than eGFP-PR8.

bioRxiv preprint doi: https://doi.org/10.1101/2023.10.31.564888; this version posted November 2, 2023. The copyright holder for this preprint (which was not certified by peer review) is the author/funder, who has granted bioRxiv a license to display the preprint in perpetuity. It is made available under aCC-BY 4.0 International license.

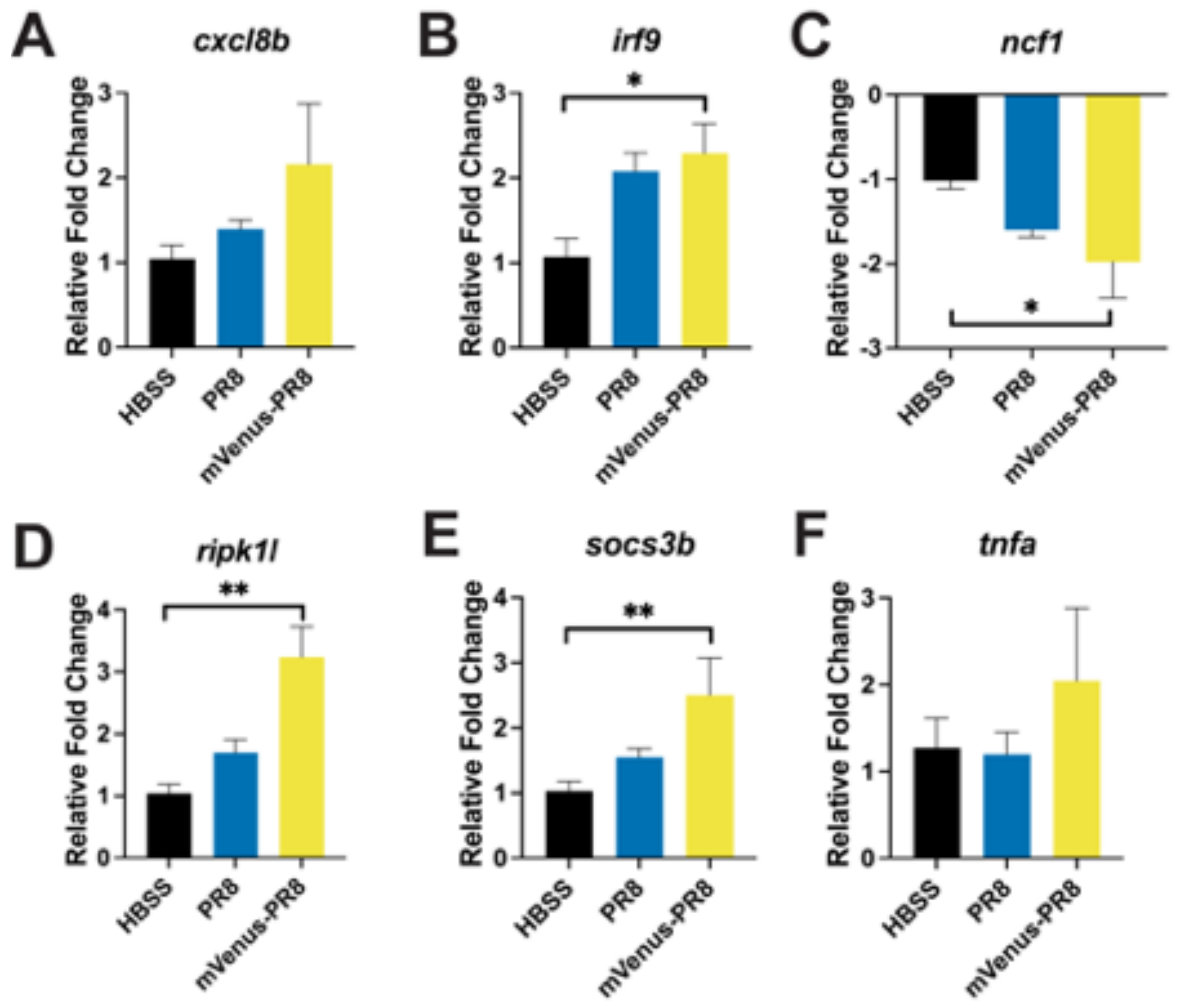

Supplemental Figure S2: Expression of candidate genes at 24 hpi. Relative fold change of six genes using qRT-PCR (*n* = 4): A) *cxcl8b*; B) *irf9* had increased expression with mVenus-PR8 infection (adjusted p-value = 0.0281); (C) *ncf1* had decreased expression with mVenus-PR8 infection (adjusted p-value = 0.0285); (D) *ripk1l* had increased expression with mVenus-PR8 infection (adjusted p-value = 0.0065); (E) *socs3b* had increased expression with mVenus-PR8 infection (adjusted p-value = 0.0088); and (F) *tnfa*.

bioRxiv preprint doi: https://doi.org/10.1101/2023.10.31.564888; this version posted November 2, 2023. The copyright holder for this preprint (which was not certified by peer review) is the author/funder, who has granted bioRxiv a license to display the preprint in perpetuity. It is made available under aCC-BY 4.0 International license.

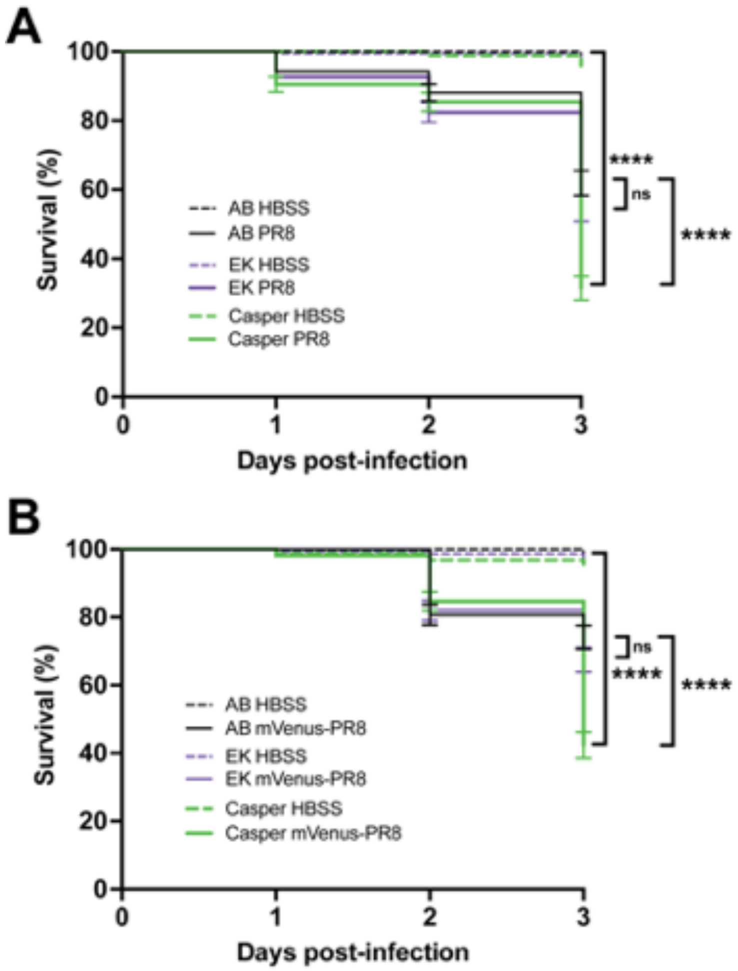

Figure S3: A) Survival of AB, EK and *casper* larvae infected with PR8 in the swimbladder at 4 dpf. Survival was lower in *casper* larvae infected with PR8 than HBSS controls ($p < 0.0001$). Survival of PR8-infected *casper* lavae was also lower than AB PR8-infected larvae ($p < 0.0001$). B) Survival of AB, EK and *casper* larvae infected with mVenus-PR8 in the swimbladder at 4 dpf. Survival was lower in *casper* larvae infected with mVenus-PR8 than HBSS controls ($p < 0.0001$). Survival of mVenus-PR8 -infected *casper* lavae was also lower than AB PR8-infected larvae ($p < 0.0001$).

bioRxiv preprint doi: https://doi.org/10.1101/2023.10.31.564888; this version posted November 2, 2023. The copyright holder for this preprint (which was not certified by peer review) is the author/funder, who has granted bioRxiv a license to display the preprint in perpetuity. It is made available under aCC-BY 4.0 International license.

807

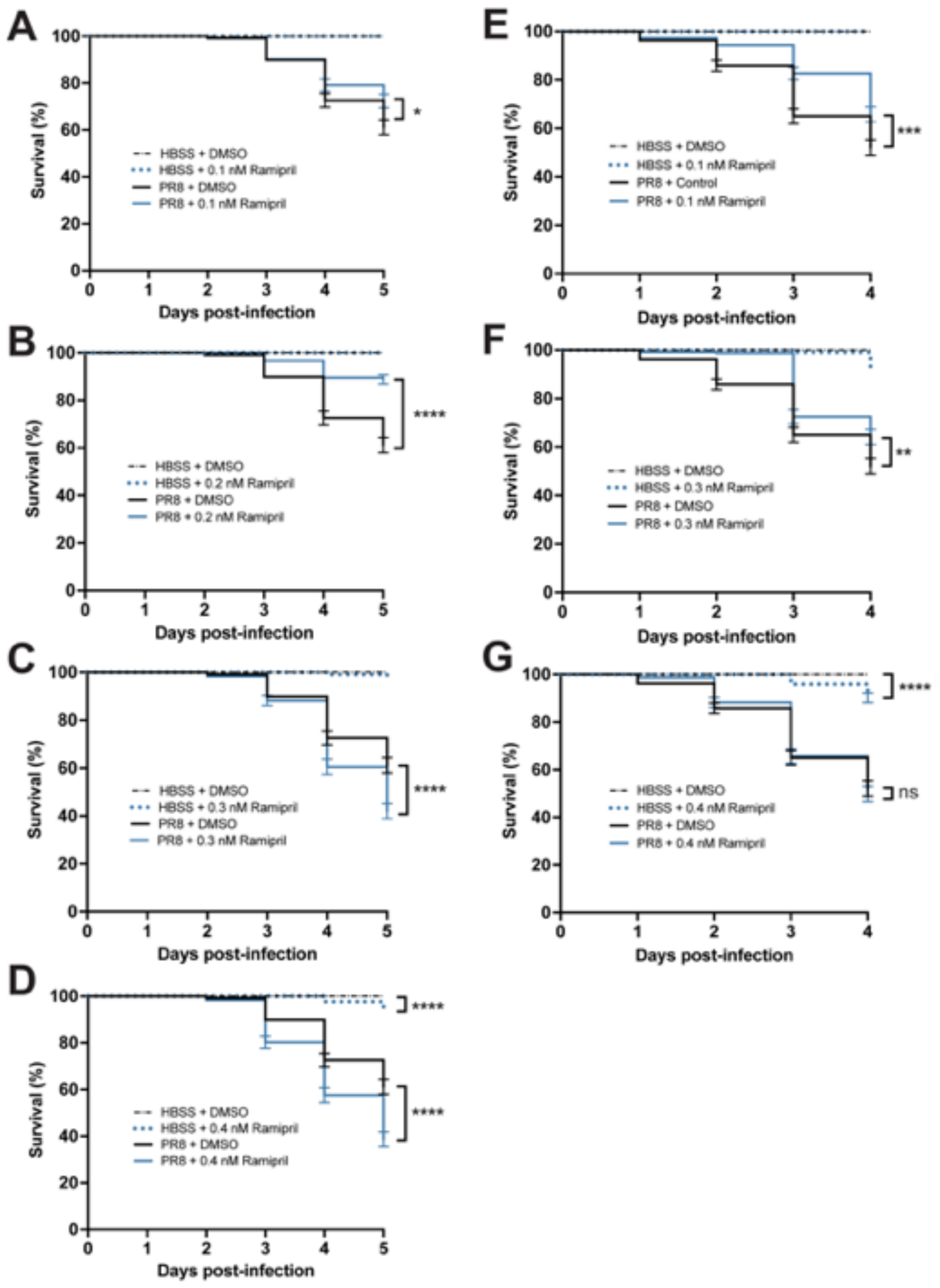

Figure S4: Survival of AB larvae infected with PR8 and treated with DMSO (control) or ramipril. A-D) Survival of larvae infected at 2 dpf and the treated with 0.1, 0.2, 0.3 or 0.4 nM MDIVI-1. Survival was higher in PR8-infected larvae with ramipril exposure at 0.1 nM ($p = 0.0157$), 0.2 nM, 0.3 nM and 0.4 nM ($p < 0.0001$) than DMSO controls. Survival was lowered with 0.4 nM ramipril exposure in HBSS controls ($p < 0.0001$). E-G)) Survival of larvae infected at 3 dpf and MDVI-1 exposure at 0.1 nM ($p = 0.0005$), 0.3 nM ($p = 0.0017$). Survival was lowered with 0.4 nM ramipril exposure in HBSS controls ($p < 0.0001$).

bioRxiv preprint doi: https://doi.org/10.1101/2023.10.31.564888; this version posted November 2, 2023. The copyright holder for this preprint (which was not certified by peer review) is the author/funder, who has granted bioRxiv a license to display the preprint in perpetuity. It is made available under aCC-BY 4.0 International license.

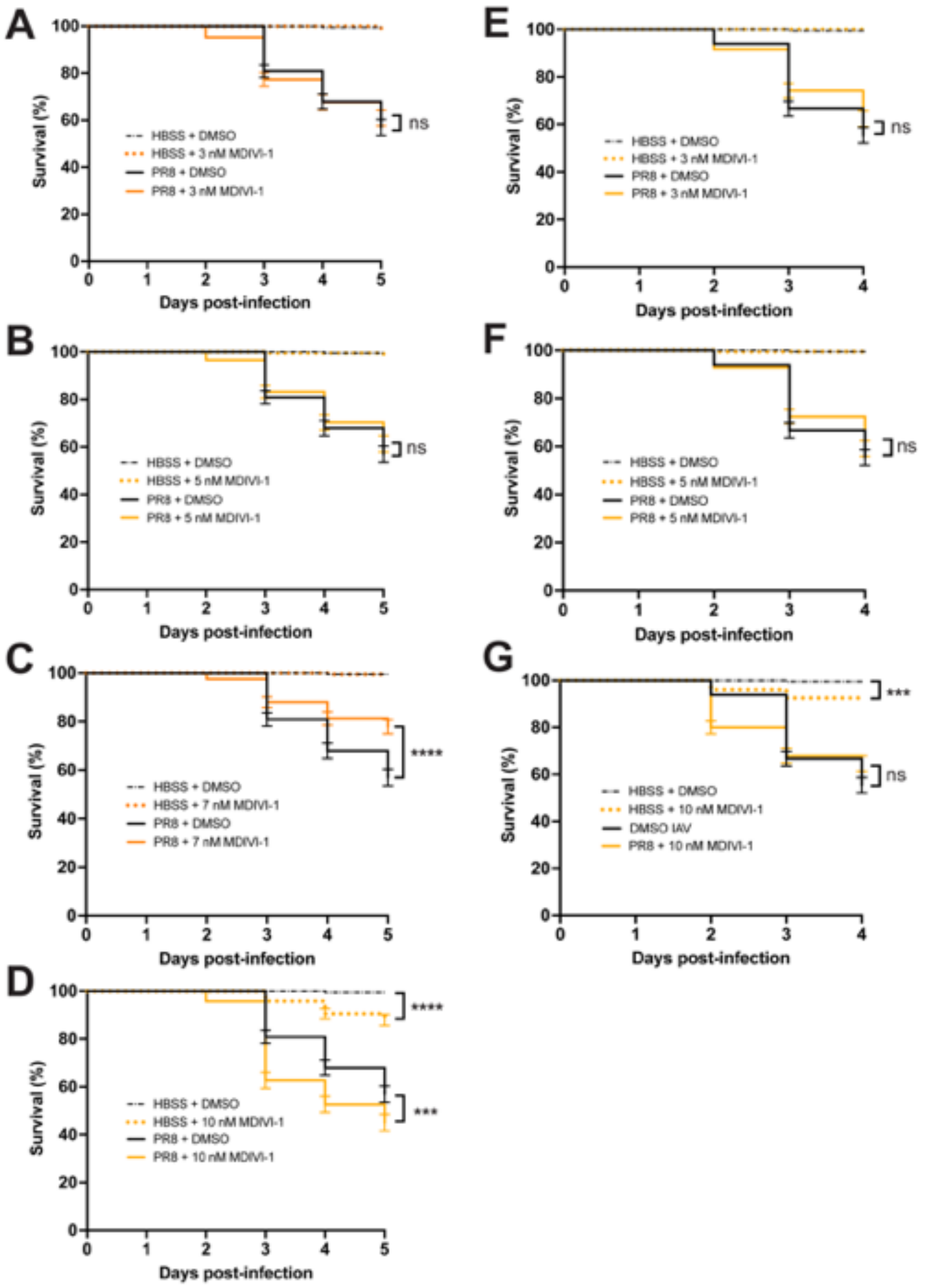

Figure S5: Survival of AB larvae infected with PR8 and treated with DMSO (control) or MDIVI-1. A-D) Survival of larvae infected at 2 dpf and the treated with 3, 5, 7 or 10 nM MDIVI-1. Survival was higher in PR8-infected larvae with 7 nM MDIVI-1 than DMSO controls ($p < 0.0001$). Survival was lowered with 10 nM MDIVI-1 exposure in HBSS controls ($p < 0.0001$) and PR8-infected larvae ($p = 0.0004$). E-G)) Survival of larvae infected at 3 dpf and the treated with 3, 5, or 10 nM MDIVI-1. Survival was lowered with 10 nM MDIVI-1 exposure in HBSS controls ($p = 0.0003$).

bioRxiv preprint doi: https://doi.org/10.1101/2023.10.31.564888; this version posted November 2, 2023. The copyright holder for this preprint (which was not certified by peer review) is the author/funder, who has granted bioRxiv a license to display the preprint in perpetuity. It is made available under aCC-BY 4.0 International license.

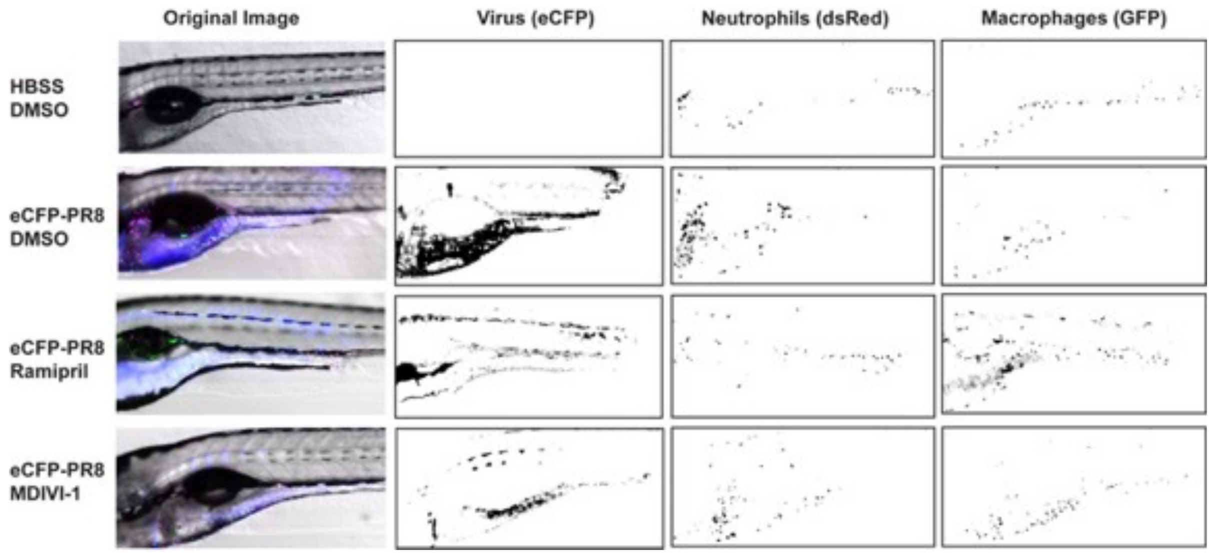

Supplemental Figure S6: Quantification of virus, neutrophils and macrophages by fluorescent confocal imaging of Tg*(mpeg1:*eGF*P;lyz:*dsRed*)* larvae at 48 hours post injection by eCFP-PR8 or HBSS following treatment by DMSO, ramipril and MDIVI-1. Representative images and masks used to quantitate the level of eCFP-PR8 virus by pixel intensities in the cyan (emission at 476 nm) channel, number of neutrophils estimated from pixel intensities in the red channel (emission at 583 nm), and number of macrophages estimated from pixel intensities in the green (emission at 510 nm) channel.
